# Informal Caregiving, Employment Status and Work Hours of the 50+ Population in Europe

**DOI:** 10.1007/s10645-018-9323-1

**Published:** 2018-05-31

**Authors:** Nicola Ciccarelli, Arthur Van Soest

**Affiliations:** CentER and Department of Econometrics and Operations Research, Tilburg University, P.O. Box 90153, 5000 LE Tilburg, The Netherlands

**Keywords:** Informal care, Labor supply, Elderly, Panel data, SHARE, J14, J22

## Abstract

Using panel data on the age group 50–70 in 15 European countries, we analyze the effects of providing informal care to parents, parents-in-law, stepparents, and grandparents on employment status and work hours. We account for fixed individual effects and test for endogeneity of caregiving using moments exploiting standard instruments (e.g., parental death) as well as higher-order moment conditions (Lewbel instruments). Specification tests suggest that informal care provision and daily caregiving can be treated as exogenous variables. We find a significant and negative effect of daily caregiving on employment status and work hours. This effect is particularly strong for women. On the other hand, providing care at a weekly (or less than weekly) frequency does not significantly affect paid work. We do not find evidence of heterogeneous effects of caregiving on paid work across European regions.

**Electronic supplementary material:**

The online version of this article (10.1007/s10645-018-9323-1) contains supplementary material, which is available to authorized users.

## Introduction

Informal caregiving refers to unpaid care provided by family members and friends, to individuals who are temporarily or permanently unable to function independently. Such care is currently the most common source of long-term care (see Costa-Font et al. 2016 and references therein). The ageing of industrialized countries’ populations, and notably the growing number of the very old, is increasing the need for informal caregiving and, more generally, the need for long-term care services (Costa-Font et al. 2015). Informal caregiving may affect the employment status and work hours of caregivers, since caregiving is a time and energy consuming activity that may be hard to combine with work duties. From a policy point of view, it is important to understand whether caregiving indeed has a negative impact on employment status or number of hours of paid work. For example, policies that reduce formal care opportunities or increase the costs of formal care will probably lead to more informal care, and it is important to know whether this has negative side effects on labour supply.

In this study we estimate the causal effects of caregiving on employment and work hours using static and dynamic panel data models. Since the majority of informal caregivers provide help to their elderly parents (see, e.g., Plaisier et al. 2015, pp. 267–274),^1^ our study focuses on the effects of informal care provision for parents, parents-in-law, stepparents, and grandparents. Our data come from SHARE, the Survey of Health, Ageing and Retirement in Europe, providing longitudinal information at the individual level on individuals of age 50 and over in a large set of European countries. SHARE contains rich data on participation in (and frequency of) informal care, and on employment and work hours.^2^

The simultaneous nature of decisions on caregiving and paid work activities makes the identification of causal effects challenging. Many older studies do not have the ambition to estimate causal effects. A substantial number of recent studies, however, aims at identifying the causal effect of caregiving on employment and work hours using a variety of models and identification strategies, e.g. panel data models with fixed effects, and cross sectional (or panel data) instrumental variable estimators treating caregiving as endogenous.

Past work has used parental health, parental death, and distance between parents and children as instrumental variables for caregiving by the respondent (see, e.g., Bolin et al. 2008; Van Houtven et al. 2013).^3^ In addition to these instrumental variables, we also use instruments that rely on higher-order moment conditions, following the methodology of Lewbel (2012). Since in this way we use two very different sources of (plausibly exogenous) variation in caregiving status, the results of endogeneity tests reported in this study are likely to be more informative than those of endogeneity tests based upon essentially only one source reported in many previous studies.

Our main findings are the following. Controlling for individual time-invariant characteristics (which may affect caregiving and employment status or work hours), we find that intensive caregiving significantly reduces the probability of being employed and the number of hours of paid work. On the other hand, providing care at a weekly (or less than weekly) frequency does not significantly affect paid work. These results are confirmed in case we also control for state-dependence in employment status (or state-dependence in work hours). Moreover, the effects of intensive caregiving on employment status and work hours are stronger for females than for males. Furthermore, interacting caregiving status with dummies for geographic region of residence,^4^ we find that the effect of caregiving on paid work is homogeneous across European regions. Finally, we do not find evidence that informal care provision and daily caregiving would be endogenous with respect to employment or work hours.

The remainder of the paper is organized as follows. In Sect. 2 we briefly discuss existing studies which analyze the effects of caregiving on paid work. In Sect. 3 we describe the SHARE data and in Sect. 4 we discuss the methodology. In Sect. 5 we discuss the evidence of the effects of caregiving on employment status and work hours. Section 6 concludes.

## Literature Review 

Table 1 summarizes some recent studies on the effects of caregiving on paid work. In a nutshell, the majority of studies have found that a high frequency of caregiving implies a negative effect on paid work (employment or work hours), though there is no consensus on the size of the effect. On the contrary, low frequency caregiving (e.g., a few hours per month) does not seem to affect paid work. Moreover, there is no evidence of a significant effect of caregiving on wages.

**Table 1:** Selected studies on the effects of informal caregiving on paid work

Study	Sample	Methodology	Main results
Berecki-Gisolf et al. (2008)	Australia, 2001 & 2004, women	Transition models	For women in paid employment in 2001, continuing or starting informal care (at least 7 hours per week) decreases the probability of being employed in 2004
Bolin et al. (2008)	11 European countries (SHARE data), 2004, age 50–64	Cross-section & instrumental variables	Informal care for one of the parents living outside the household decreases labor market participation by 22-percentage-points (p-value = 0.11). Insignificant effect on work hours or wages
Carmichael et al. (2010)	United Kingdom, 1991–2005; men, age 20–65; women, age 20–60	Transition models	Transition to providing care (at least 20 hours per week) more than doubles the chance to stop working
Ciani (2012)	13 European countries, 1994–2001; men, age 40–64; women, age 40–59	Cross-section & instrumental variables; **individual fixed effects & instrumental variables**	IV without fixed effects: strong negative impact of informal care on labor market participation (ie., 24-percentage-points decrease). IV with fixed effects: small and insignificant effects of informal care
Crespo and Mira (2014)	11 European countries (SHARE data), 2004 & 2006; women, age 50–60	**individual fixed effects & instrumental variables**	IV with fixed effects: for women that provide daily caregiving because of parental disability, daily caregiving implies a 45–65% decrease of the probability of being employed in the case of Southern Europe (LATE estimate). The effect of daily caregiving on employment is negligible in the case of Northern Europe or Central Europe
Heitmueller (2007)	United Kingdom, 1991–2002; men, age 16–64; women, age 16–59	Cross-section & instrumental variables; *non-linear panel data estimation without instrumental variables*	Cross-sectional IV estimates: large negative impact of caregiving on market participation (ie., a 34-p.-p. up to 42-p.-p. decrease). Probit estimates: intensive caregiving (ie., more than 20 h per week) decreases market participation by 12-percentage-points. No significant effects in the case of non-intensive caregiving
Jacobs et al. (2014)	Canada, 2007, age 55–69	Multinomial logit model (options: full pension, partial pension, back from retirement)	15 (or more) hours of informal care per week more than doubles the probability of full retirement with respect to being employed
Leigh (2010)	Australia, 2001–2007, age 25–64	Cross-section & *individual fixed effects without IVs*	Significant negative impact of caregiving on market participation (i.e., 5-percentage-points decrease). Marginally significant effect on work hours (i.e., -1.2 hours per week). No significant effect on hourly wages
Lilly et al. (2010)	Canada, 2002, age 45+	Cross-section without instrumental variables	Primary caregiving has a negative impact on market participation: 6.2-percentage-points decrease for women and 7.2-percentage-points decrease for men, respectively. No significant effect on work hours or wages; no significant effects of non-primary caregiving
Michaud et al. (2010)	England, 2000–2005; women, age 25–59	*Non-linear dynamic panel data model for caregiving and paid work*	Small negative effects of caregiving (for a person living either inside or outsidethe household) on paid employment
Nguyen and Connelly (2014)	Australia, 2008, working age	Multinomial logit with instrumental variables	Primary caregiving implies a negative impact on labor market participation (i.e., 12-percentage-points decrease). Insignificant effect of non-primary caregiving
Van Houtven et al. (2013)	USA, 1992–2008, age 51–78	**Individual fixed effects & instrumental variables**	For women, informal care increases the retirement probability by 2.4-percentage-points. Conditional on working in the current wave, (intensive) caregiving has negative impact on work hours. No significant effect on wages

Panel data studies that do not use instrumental variables are reported in *italics*. Panel data studies that use instrumental variables are marked in **bold**

As reported in Carmichael and Charles (1998), the effect of caregiving to elderly parents on labor supply will be the net impact of two offsetting forces. On the one hand, we expect a *substitution response*: since time is scarce, informal care responsibilities will tend to increase the caregiver’s shadow wage rate, reducing the probability of doing paid work. On the other hand, there could be a counteracting *income effect*: since caring is an expensive activity for the caregiver (see Carmichael and Charles 1998 and references therein), and since the care-receiver may not reimburse the caregiver for these costs, the expenditures associated with caring give a motive to increase earnings. Informal caregiving will reduce employment and work hours if the substitution effect dominates the income effect.

Table 1 shows that different methodologies are used to estimate the effect of caregiving on paid work. Studies based on cross-sectional data typically use instrumental variables. Parental health is often used to construct instruments for caregiving, with the argument that parental health has no effect on paid work other than through caregiving (see, e.g., Bolin et al. 2008). Some recent studies also argue that instrumental variables are unnecessary since caregiving can be considered as exogenous (Lilly et al. 2010; Jacobs et al. 2014). Longitudinal studies often look at transition probabilities (e.g., Berecki-Gisolf et al. 2008), investigating whether individuals doing informal care and paid work at time *t* have a higher probability to leave the labor market before time $t+1$ compared to individuals who are working but do not give care in period *t*. Other longitudinal studies use panel data models with individual fixed effects, thus controlling for all time-invariant confounding factors. See, e.g., Heitmueller (2007), who also compares cross-sectional IV estimates with fixed effects (non-IV) panel data estimates. The most advanced studies combine instrumental variables and panel data models; see, e.g., Ciani (2012) or Van Houtven et al. (2013). The sample period and the nature of the sample vary widely across studies. Many studies use data for one particular country (US, UK, Australia, Canada). Some only look at women who are traditionally less attached to the labor market than men and more often participate in informal care.

Summarizing the results, we can say that low-frequency caregiving often has a small and insignificant effect on paid employment. On the other hand, intensive caregiving (defined in various ways) often has a stronger effect on employment or hours of paid work than low-frequency or no caregiving. The largest effect on employment is found by Crespo and Mira (2014). They find that, for southern European women that provide daily caregiving because of parental disability, daily caregiving implies a 45–65% decrease of the probability of being employed. The largest effect on hours of paid work is found by Van Houtven et al. (2013), who find with US data that intensive caregiving reduces the working week by an average of three hours. The results also vary with methodology; Ciani (2012) shows that the impact of informal care provision on employment strongly depends on the chosen method of estimation, from about 0-percentage-points (fixed effects instrumental variables estimation) to minus 24-percentage-points (pooled instrumental variables estimation).

## Data 

The Survey of Health, Ageing and Retirement in Europe (SHARE) is a European longitudinal dataset containing information on individuals of age 50 and older and their spouses. SHARE is modeled after the US Health and Retirement Study. It is currently composed of six waves with data ranging from 2004–2005 to 2015. Wave 3 is a life history survey that did not collect the information we need and cannot be used for the current analysis. In this study, four waves of the SHARE dataset are used for a longitudinal analysis: wave 1 collected in 2004–2005, wave 2 in 2006–2007, wave 4 in 2011–2012, and wave 5 in 2013.^5^

The countries included for each wave are listed in Table 9 in the Appendix. Since we use panel data techniques, we focus on countries that are present in at least two waves.^6^ Furthermore, we do not use data for Israel since interview years for Israel differ from those of the remaining countries. Data for (respondents living in) ten countries are included for each of the four waves: Austria, Belgium, Denmark, France, Germany, Italy, Netherlands, Spain, Sweden, and Switzerland. Additionally, we use data for Greece (waves 1,2), Czech Republic (waves 2,4,5), Poland (waves 2,4), Slovenia (waves 4,5), and Estonia (waves 4,5). In our analysis, we focus on the age group 50–70. SHARE has some spouses younger than 50 years old, but since this group is not representative for all those younger than 50, we discard these observations in the analysis. Since the retirement age has been increasing across Europe in recent years and more and more people retire after age 65, we chose the upper threshold of 70 years of age.

SHARE is multidisciplinary, providing information on all relevant domains of the lives of the 50+ population. The most relevant information for our purposes is on labor market position and retirement, social support (including informal care), health, demographics, and family background (e.g., number of living parents).

*Employment Status and Weekly Work Hours* As dependent variables, we created an employment (employee or self-employed) dummy on the basis of a survey question on occupational status, and the variable hours of paid work using a survey question on usual hours of paid work including unpaid or paid overtime.^7^ We set hours of paid work equal to zero for individuals that are not currently employed. See Table 2 for the wordings of the questions and descriptive statistics by country.^8^

Employment rates vary substantially across countries, from around 50% for Switzerland and the Scandinavian countries to less than 30% for men and even less for women in Italy, Poland and Slovenia. This partially reflects differences in (early) retirement arrangements (e.g., see Schils 2008). The standard amount of weekly work hours in Europe is around 40; including overtime, the sample average of weekly hours (conditional on being employed) is 41.19 for males and 33.62 for females. The variation across countries is much larger for women than for men. The Netherlands has a particularly low sample mean for women, reflecting the fact that the large majority of Dutch women work part-time.

**Table 2:** Descriptive statistics on variables for paid work

	Participation in paid work (employment dummy) $^{\dagger }$				Weekly hours of paid work if employed employed $^{\dagger \dagger }$					
	Males		Females		Males			Females		
	Obs.	Mean	Obs.	Mean	Obs.	Mean	SD	Obs.	Mean	SD
	(1)	(2)	(3)	(4)	(5)	(6)	(7)	(8)	(9)	(10)
*Northern Europe*										
Denmark	945	0.55	1033	0.47	515	39.10	10.60	477	34.35	8.56
Sweden	875	0.53	1031	0.48	465	40.40	10.13	490	36.44	8.86
*Western Europe*										
Austria	899	0.31	1248	0.22	280	42.77	11.34	275	32.95	13.64
Belgium	1656	0.36	1799	0.30	591	41.48	12.48	544	32.00	13.62
France	1209	0.34	1600	0.35	408	40.94	12.00	559	34.53	11.72
Germany	687	0.34	838	0.37	232	43.36	11.15	311	30.87	13.28
Netherlands	951	0.41	1353	0.33	391	37.27	10.54	451	24.89	11.96
Switzerland	852	0.59	1047	0.53	502	43.08	12.69	545	29.21	14.64
*Southern Europe*										
Greece	438	0.59	472	0.26	257	44.28	18.60	117	38.40	17.11
Italy	824	0.28	1329	0.17	226	40.08	10.72	227	34.07	11.41
Spain	635	0.37	1017	0.23	232	41.02	13.65	228	34.09	14.56
*Eastern Europe*										
Czech Republic	629	0.31	1322	0.21	194	42.82	10.69	284	41.01	11.47
Estonia	559	0.45	1095	0.48	247	40.87	9.45	521	37.82	9.66
Poland	238	0.26	406	0.12	61	44.39	10.25	47	39.62	10.72
Slovenia	394	0.26	486	0.21	99	42.09	8.12	99	40.64	8.00
Total	11791	0.40	16076	0.32	4700	41.19	11.98	5175	33.62	12.69

SHARE 2004–2013; ages 50–70. Estimation sample for static model in first differences (Table 5)

$^{\dagger }$The employment dummy is based upon the question “In general, which of the following best describes your current employment situation?” (a) Employed or self-employed, (b) unemployed, (c) permanently sick or disabled, (d) homemaker, (e) retired, (f) other (i.e., rentier, living off own property, student, doing voluntary work)”. It is equal to 1 if the respondent answers “employed or self-employed”, 0 otherwise

$^{\dagger \dagger }$Weekly work hours conditional on being employed are based upon the question “Regardless of your basic contracted hours, how many hours a week do you usually work in this job, excluding meal breaks but *including any paid or unpaid overtime*?”

*Variables for Informal Care Provision* We use two variables for caregiving: (a) a dummy variable for informal care provision, taking the value 1 if the respondent helps parents, parents-in-law, stepparents or grandparents (henceforth ***“parents”***) that live in another household, and 0 otherwise; (b) a dummy variable for daily caregiving, taking the value 1 if the respondent provides informal care at daily or almost daily frequency for a “parent” that lives in another household, and 0 otherwise.^9^ We only use data on individuals that are family respondents, since questions on informal care provision are asked to non-family-respondents in waves 1 and 2 only.

The question related to informal care provision for individuals that live outside the household has changed somewhat over time. In waves 1 and 2, there is a detailed breakdown of whether the help was given for (a) personal care (e.g., dressing), (b) practical household help (e.g., shopping), (c) help with paperwork (e.g., filling out forms).^10^ In waves 4 and 5, respondents are simply asked whether they have given personal care or practical household help to someone outside the household, and there is no follow-up question regarding the type of care provided by the respondent.^11^ Therefore, in order to construct a consistent measure of informal care provision, we define informal caregiving as help for *personal care* or *practical household help*. If the respondent reported that (s)he provided help, a follow-up question asked who was the recipient, a relative (e.g., a child, a parent), a neighbor, or a friend. This exercise was repeated for up to three different recipients of informal care. We generated a dummy variable equal to 1 if the respondent provided personal care or practical household help for (at least) one “parent” living in another household, 0 otherwise, denoted as ***“informal caregiving”*** from now on.

Summary statistics on informal caregiving for male and female respondents (in the age group 50–70) by country are presented in the left-hand panel of Table 3. In the complete sample, 11.7% of all males and 13.5% of all females provide informal care to a “parent” living in another household. In all countries, females are more likely to provide informal care than males. The prevalence of informal caregiving is lowest in Greece and Poland and highest in Denmark.

**Table 3:** Descriptive statistics on variables for caregiving

	Informal caregiving$^{\dagger }$				Daily caregiving$^{\dagger \dagger }$			
	Males		Females		Males		Females	
	Obs.	Mean	Obs.	Mean	Obs.	Mean	Obs.	Mean
	(1)	(2)	(3)	(4)	(5)	(6)	(7)	(8)
*Northern Europe*								
Denmark	945	0.189	1033	0.204	945	0.008	1033	0.010
Sweden	875	0.151	1031	0.166	875	0.005	1031	0.010
*Western Europe*								
Austria	899	0.080	1248	0.097	899	0.032	1248	0.038
Belgium	1656	0.156	1799	0.174	1656	0.023	1799	0.042
France	1209	0.118	1600	0.158	1209	0.026	1600	0.040
Germany	687	0.106	838	0.142	687	0.020	838	0.035
Netherlands	951	0.128	1353	0.132	951	0.008	1353	0.019
Switzerland	852	0.112	1047	0.159	852	0.011	1046	0.024
*Southern Europe*								
Greece	438	0.041	472	0.057	438	0.007	472	0.030
Italy	824	0.102	1329	0.120	824	0.047	1329	0.075
Spain	635	0.055	1017	0.072	635	0.020	1016	0.035
*Eastern Europe*								
Czech Republic	629	0.132	1322	0.134	629	0.049	1321	0.061
Estonia	559	0.107	1095	0.128	559	0.029	1095	0.034
Poland	238	0.025	406	0.071	238	0.004	406	0.037
Slovenia	394	0.056	486	0.095	394	0.036	486	0.051
Total	11791	0.117	16076	0.135	11791	0.022	16073^*^	0.037

SHARE 2004–2013; ages 50–70. Estimation sample for static model in first differences (Table 5)

$^{*}$Three observations are missing

$^{\dagger }$Informal caregiving is a dummy variable taking value 1 for respondents that helped a “parent” that lives in another household, 0 otherwise

$^{\dagger \dagger }$Daily caregiving is a dummy variable taking value 1 for respondents that helped a “parent” at daily (or almost daily) frequency, 0 otherwise, constructed using the question: “How often in the last year did you care for this person?” with possible answers (a) almost every day, (b) almost every week, (c) almost every month, (d) less often

For each extra-residential recipient of informal caregiving, the respondent was asked whether care to this recipient was given (a) daily or almost every day, (b) almost every week, (c) almost every month, (d) less often (than at monthly frequency). We define a dummy for daily or almost daily caregiving as 1 if daily or almost daily care was given to at least one “parent” that lives in another household, 0 otherwise (henceforth ***“daily caregiving”***).^12^ The frequencies of (almost) daily care provision by gender and country are given in the right-hand panel of Table 3. Only a minority of informal care providers give informal care almost daily, particularly among males and in the Scandinavian countries. Relatively high rates of almost daily informal care are found in Italy and the Czech Republic. Overall, the participation rates in (almost) daily informal care are 2.2% for men and 3.7% for women aged 50–70.

### Control Variables

Independent variables (used as controls in the regression models) include the standard demographics age, age squared, marital status, number of children, and household size.^13^ See Table 10 in the Appendix for descriptive statistics. Almost 64% of all respondents are married. The average number of living children is 2.06, but the average household size of 2.12 shows that only a small minority of them live in the same household as their parent. Informal care decisions can depend on household composition since they are often negotiated in a family context (Heitmueller 2007, p. 538).

## Methods 

We use static and dynamic linear panel data models with fixed individual specific effects.^14^ The static model can be written as

1 $$\begin{aligned} y_{it} = x_{it}' \beta + \alpha _i + \epsilon _{it}, (i=1,..., N; t=1,..., T). \end{aligned}$$

Here $$i =1, \ldots ,N$$ and $$t = 1,\ldots ,T$$ denote the individual and the wave, respectively; $$y_{it}$$ is the employment dummy or the number of work hours. $$\beta $$ is a *K*-dimensional vector of unknown parameters; the *K*-dimensional vector $$ x_{it}$$ contains the explanatory variables (including wave fixed effects). $$\alpha _i$$ varies across individuals and is fixed over time for the same individual. To eliminate unobserved time-invariant individual heterogeneity, the model is estimated in first differences,^15^ defining $$\Delta y_{it} = y_{it} - y_{i,t-1}$$, etc.:

2 $$\begin{aligned} \Delta y_{it} = \Delta x_{it}' \beta + \Delta \epsilon _{it}, (i=1,..., N; t=2,\ldots ,T). \end{aligned}$$

The static model with strictly exogenous explanatory variables assumes that $E[\Delta x_{it} \Delta \epsilon _{it}] = 0$ for $t = 2, \ldots , T$. This model can be estimated using ordinary least squares (OLS) on the equation in first differences (2) (henceforth the *FD estimator*). If, however, $\Delta y_{it}$ has a causal effect on $\Delta x_{it}$ (reverse causality), then the FD estimator is inconsistent. Moreover, if (time-varying) omitted variables are correlated with both $\Delta x_{it}$ and $\Delta y_{it}$, then the FD estimator is also inconsistent. To account for this potential problem, the static model with potentially endogenous $\Delta x_{it}$ can be estimated using a first difference (generalized) instrumental variables estimator (FDIV), provided strictly exogenous instruments $z_{it}$ (or $\Delta z_{it}$) are available. We use several instruments that have been exploited in the existing literature; they will be discussed below in detail. These instruments rely on the moment condition $E[\Delta z_{it} \Delta \epsilon _{it}] = 0$. Moreover, we use instruments that rely on higher-order moment conditions, following Lewbel (2012). We use robust standard errors, clustered at the individual level.

In addition, we use dynamic linear panel data models with fixed individual specific effects. The dynamic panel data model can be expressed in first differences as

3 $$\begin{aligned} \Delta y_{it} = \gamma \Delta y_{i(t-1)} + \Delta x_{it}' \beta + \Delta \epsilon _{it}, (i=1,..., N; t=3,\ldots ,T), \end{aligned}$$

where $\Delta y_{i(t-1)}$ is the *state dependence variable* (in first differences) and $\gamma$ is the state dependence parameter. The dynamic panel data models are estimated with the Generalized Method of Moments (GMM), where the moments depend on the assumptions on $\epsilon _{it}$ (and $x_{it}$). The assumption that $\epsilon _{it}$ is independent of everything before *t* implies that $y_{is}$, with $(s = 1,\dots ,t-2)$, is independent of $\Delta \epsilon _{it}$. This leads to moments with instruments for the state dependence variable (Arellano and Bond 1991).^16^ Under the weaker assumption that $\epsilon _{it}$ is independent of everything in time period $t-2$ or earlier, higher order lags such as $y_{is}$, with $s \le t-3$, need to be used as instruments. Moreover, strictly exogenous instruments $z_{it}$ (or $\Delta z_{it}$) can be used for endogenous variables contained in $\Delta x_{it}$. Since the residuals may be heteroscedastic or arbitrarily correlated over time, we use cluster-robust standard errors, clustered at the individual level. The estimator for the dynamic panel data model in differences (3) is denoted as the *AB (Arellano Bond) estimator*.^17^ Finally, since the estimation of dynamic panel data models relies on the assumptions on $\epsilon _{it}$ (see above), we test for serial correlation of the error term in levels ($\epsilon _{it}$), using the test proposed in Section 10.6.3 of Wooldridge (2002).^18^

### Instrumental Variables for Informal Caregiving and for Daily Caregiving 

To allow (and test) for endogeneity of the caregiving variables, we use the standard moment condition $E[\Delta z_{it} \Delta \epsilon _{it}]=0$, as well as higher-order moment conditions using so-called Lewbel instruments (see Lewbel 2012) exploiting heteroskedasticity.

*Parental Survival Status and Health, and Distance from Parental Residence* We first describe the instrumental variables that are based on the moment condition $E[\Delta z_{it} \Delta \epsilon _{it}]= 0$. Following the literature, we constructed several instrumental variables for (daily or almost daily) caregiving. The most common instruments used in the literature are based upon health and survival status of parents or health of household members (see, e.g., Bolin et al. 2008; Ciani 2012; Heitmueller 2007; Van Houtven et al. 2013). In our set of instrumental variables ($z_{it}$), we therefore include: (1) a dummy variable equal to 1 if the respondent’s mother is dead in wave *t*, 0 otherwise; (2) a dummy equal to 1 if the respondent’s father is dead in wave *t*, 0 otherwise; (3) a dummy equal to 1 if the respondent’s mother (is alive and) has poor or very poor health in wave *t*, 0 otherwise; (4) a dummy equal to 1 if the respondent’s father (is alive and) has poor or very poor health in wave *t*, 0 otherwise.^19^ Moreover, following Bolin et al. (2008), we include an instrument based upon the (geographical) distance between respondent and potential care recipient: (5) a dummy equal to 1 if the respondent’s mother (is alive and) lives less than 1 kilometer away from the respondent in wave *t*, 0 otherwise. We also constructed an instrumental variable that is based on distance between the father’s residence and the respondent’s residence; however, but this was not added since it proved to be a very weak instrument.^20^

Existing studies conclude that these instrumental variables are likely to be valid. Coe and van Houtven (2009) find that *parental health* does not have a direct effect on the respondent’s health or depressive symptoms, implying that parental death is unlikely to directly affect the respondent’s work behavior via the bereavement effect. It is likely that the findings reported in Coe and van Houtven (2009) also apply in our study, since we use a similar panel data framework as Coe and van Houtven (2009). In a cross-sectional framework, it is often argued that *parental* *health* may affect the work behavior of adult children via the transmission of health-related genetic characteristics (e.g., see the discussion in Van Houtven et al. 2013, p. 243). Since we account for fixed effects, however, this problem does not occur, since genetic characteristics are time-invariant and will be filtered out. *Distance from parental residence* is related to the time cost of providing informal care. With smaller distance between the informal caregiver and the care-receiver, time costs for the former will decrease, which in turn may increase the amount of informal care provided. Since we control for individual fixed effects that capture time-invariant preferences for work and time-invariant preferences related to distance from the parental residence, the instrument “distance from the mother’s residence” is likely to affect the respondent’s employment status only through its effect on caregiving.

*Heteroscedasticity-based Instrumental Variables* The *second source of identification* exploits higher-order moment conditions. The first aim of adding this second source of identification is to increase the (first-stage) strength of the instrument set. Moreover, having two very different types of instruments helps to increase the reliability of instrumental variables estimates; see Murray (2006), specifically, the section “Use Alternative Instruments”. As shown in Lewbel (2012), variables *Z* that are correlated with heteroscedasticity of the first-stage equation and that are uncorrelated with the product of the error terms of the first-stage and second-stage equations can be exploited to construct interactions of $Z-\bar{Z}$ with the residuals from the first stage equation explaining the endogenous regressor as instruments. The first condition can be easily tested through a Breusch-Pagan test for heteroskedasticity. *Z* can contain control variables, or variables that are excluded from the regression model, or both (see page 70 of Lewbel 2012).

We use for *Z* (1) respondent’s age, (2) respondent’s household size, (3) a time dummy for the second wave, which are all significantly correlated with the heteroscedasticity of the error term of the first stage equation at the 5% significance level.

In our context of estimation in first differences, the heteroscedasticity-based instruments are constructed using the following procedure: (1) we estimate the first-stage equation $\Delta Caregiving_{it} =$ $\Delta Controls_{it}' \beta +$ $+ \Delta \epsilon _{2it}$ by OLS, where $\Delta \epsilon _{2it}$ is the first-stage error term in first differences, and where $Controls_{it}$ include both control variables and wave fixed effects; (2) we save the first-stage residuals $\widehat{\Delta \epsilon _{2it}}$; (3) we construct $[\Delta Z_{it} - E(\Delta Z_{it})]$, where $\Delta Z_{it}$ is a vector of random variables in first differences that is (assumed to be) uncorrelated with the product of the error terms $(\Delta \epsilon _{1it} \Delta \epsilon _{2it})$; (4) we generate the heteroscedasticity-based instruments, $[\Delta Z_{it} - E(\Delta Z_{it})] \widehat{\Delta \epsilon _{2it}}$.

## Results 

We study the effects of informal caregiving (at any frequency) and daily caregiving on participation in paid work (an employment dummy) and paid work hours. In order to analyze the sensitivity of the estimates for the model assumptions, we present the main results for a number of (static and dynamic) panel data models. We first analyze the effects of (informal and daily) caregiving on employment and on work hours using the complete sample. Finally, we also investigate whether the impact of informal caregiving and daily caregiving on paid work variables differs by gender or across European regions.

### The Effects of Informal Caregiving and Daily Caregiving on Employment

We first present estimates using techniques that do not exploit instrumental variables for informal caregiving and daily caregiving. Subsequently, we discuss estimates using static and dynamic panel data models, with the instruments for caregiving presented in Sect. 3 and using lags as instruments for the lagged dependent variable.

**Table 4:** The effects of caregiving on employment—OLS, FD, (Static) FDIV, and AB (Arellano Bond) estimates

	(1)	(2)	(3)	(4)	(5)	(6)	(7)	(8)
	OLS	OLS	FD	FD	FDIV	FDIV	AB	AB
					Second stage	Second stage	Second stage	Second stage
*Dependent variable: employment*								
Informal caregiving	0.057***	–	$-$ 0.005	–	0.028	–	$-$ 0.016	–
	(0.008)		(0.006)		(0.038)		(0.016)	
Daily caregiving	–	$-$ 0.107***	–	$-$ 0.027**	–	$-$ 0.024	–	$-0.065$**
		(0.015)		(0.011)		(0.045)		(0.025)
Employment$_{i,t-1}$	–	–	–	–	–	–	0.509***	0.509***
	–	–	–	–	–	–	(0.026)	(0.026)
Individual FE?	No	No	Yes	Yes	Yes	Yes	Yes	Yes
Wave FE?	Yes	Yes	Yes	Yes	Yes	Yes	Yes	Yes
Controls?$^{\dagger }$	All	All	All	All	All	All	All	All
Observations	27,867	27,864	27,867	27,864	27,867	27,864	7609	7609
N (persons)	20,954	20,952	20,954	20,952	20,954	20,952	5321	5321
Adjusted $R^2$	0.283	0.283	0.010	0.010	–	–	–	–
*Test results*								
F-statistic (excluded IVs)	–	–	–	–	28.676	11.050	1037.908	1037.175
Hansen J test (p-value)	–	–	–	–	0.219	0.114	0.378	0.372
Hausman test (p-value)	–	–	–	–	0.337	0.721	0.000	0.000
*Test of serial correlation of the error term in differences:* $\Delta \epsilon _{it} = \rho \Delta \epsilon _{i,t-1} + error_{it}$								
p-value (test: $\widehat{\rho } =-0.5$)	–	–	–	–	–	–	0.502	0.519

***$p<0.01$, **$p<0.05$, *$p<0.10$. Robust standard errors, which are clustered at the individual level, are reported in parentheses. “OLS”, “FD”, “FDIV”, “AB” refer to (pooled) ordinary least squares, first difference, first difference IV, Arellano Bond estimators, respectively. See Table 13 (electronic supplementary materials) for more details on OLS and FD estimates. See Tables 14 and 15 (electronic supplementary materials) for more details on FDIV and AB estimates, respectively

$^{\dagger }$Controls include age, age squared, marital status (i.e., a dummy variable equal to 1 for married persons, 0 otherwise), number of children and household size

#### OLS and First Difference Estimates

Pooled OLS and FD (first difference) estimates for the effects of the caregiving variables on employment are reported in Columns 1–4 of Table  4.^21^ Using the pooled OLS estimator, we find that the coefficient on informal caregiving is positive and significant at the 5% level, suggesting that people who are active on the labor market also tend to give informal care, but not necessarily reflecting any causal relationship (see Column 1 of Table 4). Controlling for individual fixed effects with the FD estimator reverses the sign of the coefficient on informal caregiving, and makes the coefficient statistically insignificant at the 5% level (see Column 3 of Table 4).

We find that providing care at a (almost) daily frequency negatively affects the employment probability, both in the case that we do not control for individual fixed effects (see Column 2), and in the case that we control for individual fixed effects (see Column 4). In the latter case, the coefficient on daily caregiving is significant at the 5% level, suggesting that it is hard to combine daily caregiving and work. The estimate reported in Column 4 implies that providing care at a (almost) daily frequency reduces the probability of being employed by 7.6% (2.7-percentage-points) on average. By and large, these results are in line with earlier results. For example, Lilly et al. (2007) found that “only those heavily involved in caregiving are significantly more likely to withdraw from the labor market than non-caregivers.” These estimates may still not reflect causal effects, e.g., due to reverse causality which would make the caregiving variable endogenous. We test whether the caregiving variables are exogenous by comparing FD estimates with first difference instrumental variables (FDIV) estimates, using the instrumental variables presented in Sect. 4.1.

#### First Difference IV Estimates

We present (second-stage) FDIV estimates of static panel data models in Columns 5–6 of Table  4. As shown in Column 5 of Table 4, using the set of eight instrumental variables described in Sect. 4.1, we find that the null hypothesis of exogeneity of informal caregiving is not rejected at the 5% significance level (Hausman test’s p-value = 0.337). Moreover, the instrumental variables are jointly strong (F-statistic = 28.68),^22^ and the over-identifying restrictions are not rejected at the 5% significance level (Hansen test’s p-value = 0.219). The latter result indicates that the instrumental variables are likely to be exogenous. Finally, in line with the estimate from the first difference regression (see Column 3), we find that the second-stage coefficient on informal caregiving is insignificant (see Column 5). Based on the results from first difference and first difference IV estimations, we can conclude that low-intensity informal caregiving does not exert a negative effect on employment.

If the instrumented regressor is daily caregiving, we also find that exogeneity is not rejected at the 5% significance level (Hausman test’s p-value = 0.721); see Column 6 of Table 4. This suggests that daily caregiving is exogenous with respect to the error term in the employment equation. Moreover, the instrumental variables are jointly strong to predict daily caregiving in the first-stage equation (F-statistic = 11.05),^23^ and the instruments are likely to be exogenous since the over-identifying restrictions test does not reject at the 5% significance level (Hansen test’s p-value = 0.114).

In line with the estimate from the first difference regression (see Column 4 of Table 4), we find that the effect of daily caregiving on employment is negative. Moreover, the magnitude of the (second-stage) coefficient on daily caregiving is almost identical to the magnitude of the corresponding coefficient obtained with the first difference regression (see Column 4 and Column 6 of Table 4). These results suggest that the substitution effect arising from daily informal care provision dominates the income effect, i.e., daily caregiving exerts a negative effect on employment.^24^

To conclude, in line with some studies reported in Sect. 2, we find that the caregiving variables are exogenous with respect to the error term on employment in the case that we control for individual fixed effects, wave fixed effects, and control variables. Additionally, considering the results of first difference and first difference IV estimations, we find evidence of a disemployment effect of daily caregiving, while providing informal care at any frequency does not imply a disemployment effect. These results are in line with the existing literature using panel data for other countries, such as Heitmueller (2007). He uses British data and finds an insignificant effect of caregiving but a significant effect of intensive caregiving, both in cross-section IV models and in standard fixed effects panel data models.

#### Arellano Bond Estimates

We present (second-stage) Arellano Bond estimates of dynamic panel data models in the last two columns of Table 4.^25^ Since we control for lagged dependent variable (see Eq. (3)), the sample size shrinks from around 27,860 observations (static model) to 7609 observations in the case of dynamic models. Additionally, due to the inclusion of the lagged dependent variable in the regression model, respondents included in the sample for dynamic models are approximately 2 years older than respondents that are included in the sample for static models. Since the Hausman tests suggest that caregiving variables can be treated as exogenous variables also in the dynamic models,^26^ we present the AB estimates that do not use instruments for the caregiving variables, using $employment_{i(t-2)}$ and $employment_{i(t-3)}$ as instruments for the state dependence variable in differences ($\Delta employment_{i(t-1)}$).

Since these instruments rely on the assumption on serial correlation of the error term in levels ($\epsilon _{it}$), we first test for serial correlation in the error term. We do not reject the hypothesis that the error term in levels is not serially correlated at the 5% significance level (see the last row of Table 4), implying that the second and third lags of employment can be used as instruments for the state dependence variable ($\Delta y_{i(t-1)}$). Using the above-mentioned instruments for the state dependence variable ($\Delta y_{i(t-1)}$), we find that the lagged dependent variable is always highly significant (see Columns 7–8). Moreover, the state dependence parameter is close to 0.5, indicating that being employed in the previous wave increases the probability of being employed in the current wave by approximately 50-percentage-points.

We find that, in line with the (static) FD estimate, the coefficient on informal caregiving is insignificant at the 5% level (see Column 3 and Column 7, respectively). This confirms our previous finding that informal caregiving as such does not exert a negative effect on employment. On the other hand, the coefficient on daily caregiving is significant at the 5% level, and daily caregiving leads to a 6.5-percentage-points decrease (22.0% decrease)^27^ of the probability of being employed (see Column 8). In line with results from the static (FD) model, we conclude that daily caregiving exerts a strong disemployment effect.

The estimated coefficient on daily caregiving in the dynamic panel data model is much larger than the corresponding coefficient in the static (FD) model (see Column 4 and Column 8 of Table 4, respectively). For this reason, we also estimated the effect of daily caregiving on employment using the smaller sample of 7609 observations—i.e., the sample for the dynamic model—and using the (static) FD estimator; in this case, the coefficient on daily caregiving is $-0.056$ ($p=0.003$). The latter result implies that the contrasting evidence obtained with the static FD model and the dynamic panel data model can be ascribed to two main factors: (a) the inclusion of the state dependence variable in the regression model; (b) the presence of older individuals in the sample for the dynamic panel data model. Indeed, we may expect a stronger disemployment effect of daily caregiving for older individuals than for the overall sample: compared to younger individuals, older individuals may have a higher preference for dropping out of the labor market (or for retirement) when they are confronted with the need of providing daily informal care.

To summarize, we find that the assumptions of the dynamic model are supported by the data. Furthermore, the lagged dependent variable exerts a strong effect, implying that employment is strongly persistent. Omitting the state dependence variable from the regression model thus might lead to misleading conclusions. Based on these previous arguments, we prefer the dynamic panel data models to the static panel data models, as the dynamic models capture the apparent state dependence in employment.

#### Combining Daily and Non-daily Caregiving

In the previous analysis, we separately analyzed the effect of the dummy variables for informal care provision and daily care provision on employment.^28^ This choice was made for the following reason. We first tried to use both a dummy variable for *informal care provision at weekly and less than weekly frequency* (henceforth, **non-daily caregiving**), and a dummy variable for *daily caregiving*, but the instrumental variables were jointly weak for the (joint) instrumentation of these dummy variables.^29^ Since the variables for informal care provision used in the previous analysis appear to be exogenous with respect to the error term on employment (see discussion above), we present extensions of the models treating caregiving as exogenous in which non-daily caregiving and daily caregiving can affect employment differently, using the pooled OLS, (static) first difference and AB estimators;^30^ the regression results reported below account for the time-varying control variables reported in Sect. 3 and wave fixed effects.

The estimation results are presented in Table 11 in the Appendix. Pooled OLS estimates provide correlational evidence for the relationship between caregiving and employment status. Using the (static) first difference and AB estimators, we find that *non-daily caregiving* is completely insignificant (see Columns 2–3 of Table 11), i.e., low-frequency informal care does not increase the likelihood of withdrawal from the labor market. On the contrary, *daily caregiving* is statistically significant at the 5% level using both the first difference and AB estimators (see Columns 2–3 of Table 11). Moreover, using the same panel data specifications, we find that the estimated coefficient on daily caregiving is approximately the same as in the case where the control group is composed of both non-caregivers and non-daily caregivers (see Columns 2 and 3 of Table 11 in the Appendix, and see Columns 4 and 8 of Table 4, respectively).

To conclude, we find that the estimates for the effect of daily caregiving on employment status are not impacted by the choice of the control group. This implies that providing daily care to an elderly “parent” in the current wave has the same (negative) effect on employment for individuals that were non-caregivers or provided care at low frequency in the previous wave.

### The Effects of Informal Caregiving and Daily Caregiving on Work Hours

In this section, we report estimates for the effect of informal caregiving and daily caregiving on work hours. We first present pooled OLS and first difference estimates, not using instruments for informal and daily caregiving. Next, we discuss first difference instrumental variables (FDIV) and Arellano Bond (AB) estimates, using instruments for the caregiving variables and/or for the lagged dependent variable.

#### Pooled OLS and First Difference Estimates

Pooled OLS and FD estimates for the effect of caregiving on work hours are reported in Columns 1–4 of Table 5.^31^ The coefficient on informal caregiving switches from positive and statistically significant in the case of (pooled) OLS estimation to negative and insignificant in the case of first difference estimation; see Columns 1 and 3, respectively. This is similar to what we found for the employment dummy.

The OLS estimate of the effect of daily caregiving on work hours is significant and negative (see Column 2 of Table  5). In line with the results obtained for the effect of daily caregiving on employment (see Sect. 5.1), the FD estimates show that variation in daily caregiving across waves implies a significant decrease of work hours (see Column 4). The size of the effect is substantial: Daily caregiving implies a 11.8% decrease of work hours.^32^

**Table 5:** The effects of caregiving on paid work hours—OLS, FD, (Static) FDIV, and AB (Arellano Bond) estimates

	(1)	(2)	(3)	(4)	(5)	(6)	(7)	(8)
	OLS	OLS	FD	FD	FDIV	FDIV	AB	AB
					Second stage	Second stage	Second stage	Second stage
*Dependent variable: work hours*								
Informal caregiving	1.829***	–	$-$ 0.292	–	0.205	–	$-$ 1.218*	–
	(0.353)		(0.262)		(1.574)		(0.636)	
Daily caregiving	$-$	$-$ 4.757***	–	$-$ 1.553***	–	$-$ 0.758	–	$-$ 2.902***
		(0.574)		(0.448)		(1.886)		(1.057)
Work hours$_{i,t-1}$	–	–	–	–	–	–	0.482***	0.483***
	–	–	–	–	–	–	(0.028)	(0.028)
Individual FE?	No	No	Yes	Yes	Yes	Yes	Yes	Yes
Wave FE?	Yes	Yes	Yes	Yes	Yes	Yes	Yes	Yes
Controls?$^{\dagger }$	All	All	All	All	All	All	All	All
Observations	27,621	27,618	27,621	27,618	27,621	27,618	7522	7522
N (persons)	20,788	20,786	20,788	20,786	20,788	20,786	5,270	5270
Adjusted $R^2$	0.250	0.251	0.010	0.011	–	–	–	–
*Test results*								
F-statistic (excluded IVs)	–	–	–	–	28.908	10.979	840.009	839.039
Hansen J test (p-value)	–	–	–	–	0.426	0.344	0.904	0.907
Hausman test (p-value)	–	–	–	–	0.699	0.546	0.000	0.000
*Test of serial correlation of the error term in differences:* $\Delta \epsilon _{it} = \rho \Delta \epsilon _{i,t-1} + error_{it}$								
p-value (test: $\widehat{\rho } =-0.5$)	–	–	–	–	–	–	0.029	0.027

***$p<0.01$, **$p<0.05$, *$p<0.10$. Robust standard errors clustered at the individual level in parentheses. “OLS”, “FD”, “FDIV”, “AB” refer to (pooled) ordinary least squares, first difference, first difference IV, Arellano Bond estimators, respectively. See Table 16 (electronic supplementary materials) for full OLS and FD estimates. See Tables 17 and 18 (electronic supplementary materials) for more details on FDIV and AB estimates, respectively

$^{\dagger }$Controls include age, age squared, marriage status (i.e., a dummy variable equal to 1 for married persons, 0 otherwise), number of children and household size

#### First Difference IV Estimates

Since we cannot exclude the possibility that the caregiving variables are correlated with the error term on work hours, we consider first difference instrumental variables (FDIV) estimates, and compare FD and FDIV estimates in order to test for exogeneity of the caregiving variables. The instrumental variables were already described in Sect.  4.1. The (second-stage) FDIV estimates of static panel data models are presented in Columns 5–6 of Table  5. The instruments are jointly strong (F-statistic=28.91). As shown in Column 1 of Table 17 (electronic supplementary materials), the first-stage coefficients on the variables representing parental death, parental health and distance from the maternal residence are as expected. The null hypothesis of exogeneity of informal caregiving is not rejected at the 5% significance level (Hausman test’s p-value = 0.669). Moreover, the over-identifying restrictions test does not reject (Hansen test’s p-value = 0.426), suggesting that the instruments are valid. In line with the result from the first difference regression reported in Column 3, we find that the second-stage coefficient on informal caregiving is insignificant and close to zero,^33^ indicating that informal care provision does not significantly reduce the number of paid work hours.

Column 6 shows that the instrumental variables for daily caregiving are jointly strong (F-statistic=10.979).^34^ They also seem to be exogenous, since the null hypothesis of the Hansen test is not rejected at the 5% significance level (Hansen test’s p-value = 0.344). Moreover, the null hypothesis of exogeneity of daily caregiving is not rejected at the 5% significance level (Hausman test’s p-value = 0.546). The second-stage coefficient on daily caregiving is negative, confirming that daily caregiving tends to reduce the number of work hours. Due to the large standard error associated to the coefficient on daily caregiving, we find that daily caregiving is not significant at the 5% level. However, considering the results from the FD and FDIV estimations, and in particular considering the fact that we do not reject the null hypothesis of exogeneity of daily caregiving, our results lead to the conclusion that providing informal care on a daily basis reduces the number of paid work hours.

#### Arellano Bond Estimates

Second-stage estimates for dynamic panel data models are reported in the last two columns of Table  5.^35^ In unreported regressions, we found that exogeneity of the caregiving variables is not rejected in the dynamic models.^36^ We therefore do not use instruments for the caregiving variables (but we use $work hours_{i(t-2)}$ and $work hours_{i(t-3)}$ as instruments for the lagged dependent variable ($\Delta work hours_{i(t-1)}$)).

We do not reject the assumption that the error terms in levels are serially uncorrelated at the 1% significance level (see the last row of Table 5). This implies that the second and third lags of work hours can be used as instruments in the equation in first differences. Using these instruments for the lagged dependent variable, we find that the state dependence parameter is positive and highly significant (see Columns 7–8). Its point estimate is around 0.48, indicating that working an additional hour in the previous wave increases the predicted number of paid work hours in the current wave by 0.48 (hours).

Informal caregiving does not have a significant impact on work hours at the 5% significance level (see Column 7), confirming the result of the static FD model (see Column 3). Moreover, in line with the result from the static (FD) model, we find that daily caregiving significantly reduces the number of paid work hours: caring for a “parent” at daily frequency leads to a 27.7% decrease of work hours.^37^

The estimated effect of daily caregiving on work hours from the dynamic panel data model is much stronger than in the static FD model; see Column 4 and Column 8 of Table 5, respectively. We therefore also estimated the effect of daily caregiving on work hours using the static FD estimator and the smaller sample of 7522 observations—i.e., the sample for the dynamic model. From the latter regression we obtained that caring for a “parent” at daily frequency leads to a (statistically significant) 23.0% decrease of work hours.^38^ Since the latter estimate lies between the estimate from the static FD model (see Column 4) and the estimate from the dynamic panel data model (see Column 8), the contrasting evidence obtained with the static FD model and the dynamic panel data model might be explained by two factors: (a) the inclusion of the lagged dependent variable in the regression model; (b) the presence of older individuals in the sample for the dynamic panel data model (see discussion in Sect. 5.1). The age of individuals included in the sample may affect the relationship between daily caregiving and work hours. Indeed, compared to younger individuals, older individuals may have a higher preference for working fewer hours (or to drop out of the labor market) in the case that they also need to provide informal care on a daily basis.

Since the main assumption for dynamic panel data models—i.e., absence of serial correlation of the error term in levels - is not rejected at the 1% significance level, and since the state dependence parameter is strongly significant, we prefer dynamic panel data models to static panel data models.

#### Combining daily and non-daily caregiving

As an extension, we discuss estimates for models allowing for separate effects of non-daily caregiving (at weekly or less than weekly frequency) and daily caregiving on work hours. This specification implies that the control group used in the following analysis includes only non-caregivers. We report pooled OLS, first difference, and AB estimation results in Table 12 in the Appendix. Controlling for individual fixed effects through the first difference and AB estimators, we find that the coefficient on non-daily caregiving is completely insignificant (see Columns 2–3 of Table 12). This implies that, from a statistical point of view, providing informal care at weekly (or less than weekly) frequency does not imply a decrease of work hours. On the contrary, the coefficient on daily caregiving is statistically significant at the 5% level using both the first difference and AB estimators (Columns 2–3 of Table 12). Moreover, using the first difference and AB estimators, we find that the estimated effect of daily caregiving on work hours is very similar in the case that the control group includes only non-caregivers (see Columns 2–3 of Table 12), or both non-caregivers and non-daily caregivers (Columns 4 and 8 of Table 5).

### Heterogeneity by Gender

Since females are less attached to the labor market than males (see Table 2), and since females are more likely to provide daily care (and more generally informal care) to “parents” than males (see Table 3), we investigated whether the effect of daily caregiving on employment and work hours differs across genders; estimates by gender for the effect of (any type of) informal caregiving on employment and work hours are not reported (but are available from the authors), since in line with the results reported in Tables 4 and 5, the coefficient on informal caregiving is completely insignificant for both genders. Static FD estimates for the effect of daily caregiving on employment and work hours are reported in Panel A of Table 6. As shown in Columns 1–2 of Panel A, daily caregiving does not significantly affect employment or work hours for males, but significantly affects both the probability of being employed and work hours for females (see Columns 3–4 of Panel A). For females, daily caregiving implies a 10.5% decrease of the probability of being employed and a 13.1% decrease of work hours.^39^ Both effects are larger than the corresponding effects obtained for the full sample (see Sects. 5.1–5.2). A potential explanation is that labor supply of European females in the age group 50–70 is much more flexible than that of males, for example due to substitution between paid work and housework (see Hank and Juerges 2007, Table 1). Women may drop out of the labor market (or they may work fewer hours) when they have to provide daily care for a “parent”, since they may not be able to combine daily caregiving, household chores and work duties.

**Table 6:** The effects of daily caregiving on paid work by gender—FD and AB (two step GMM) estimates

	(1)	(2)	(3)	(4)
	FD	FD	FD	FD
	*Male sample*		*Female sample*	
	Dependent variables			
	Employment	Work hours	Employment	Work hours
*Panel A: FD estimates*				
Daily caregiving	$-$ 0.011	$-$ 1.851*	$-$ 0.034***	$-$ 1.410***
	(0.019)	(0.977)	(0.013)	(0.479)
Age	0.050***	2.254***	0.040***	1.364**
	(0.018)	(0.829)	(0.015)	(0.590)
Age squared	$-$ 0.001***	$-$ 0.022***	$-$ 0.0004***	$-$ 0.013***
	(0.0001)	(0.006)	(0.0001)	(0.004)
Married	$-$ 0.001	$-$ 1.292	$-$ 0.016	$-$ 1.310*
	(0.026)	(1.261)	(0.017)	(0.686)
Number of children	$-$ 0.019**	$-$ 0.602**	$-$ 0.0001	$-$ 0.149
	(0.008)	(0.299)	(0.006)	(0.217)
Household size	$-$ 0.0001	$-$ 0.036	$-$ 0.009*	$-$ 0.071
	(0.006)	(0.277)	(0.005)	(0.192)
Wave 3	$-$ 0.071***	$-$ 3.230***	$-$ 0.045**	$-$ 2.215***
	(0.022)	(1.009)	(0.018)	(0.703)
Wave 4	$-$ 0.058***	$-$ 2.415**	$-$ 0.060***	$-$ 2.830***
	(0.022)	(0.993)	(0.018)	(0.700)
Constant	$-$ 0.065***	$-$ 2.921***	$-$ 0.036*	$-$ 1.243
	(0.024)	(1.107)	(0.020)	(0.772)
Observations	11,790	11,670	16,074	15,948
N (persons)	8,883	8,795	12,069	11,991
Adjusted $R^2$	0.016	0.016	0.007	0.007

	(1)	(2)	(3)	(4)
	*Males*		*Females*	
	Dependent variables			
	Employment	Work hours	Employment	Work hours
*Panel B: AB (Arellano Bond) second-stage estimates*				
Employed$_{i(t-1)}^{\dagger }$	0.476***	–	0.521***	–
	(0.040)		(0.035)	
Work hours$_{i(t-1)}^{\dagger }$	–	0.446***	–	0.500***
		(0.039)		(0.040)
Daily caregiving	$-$ 0.035	$-$ 3.544	$-$ 0.081***	$-$ 2.640***
	(0.046)	(2.579)	(0.030)	(0.920)
Age	$-$ 0.256***	$-$ 8.799***	$-$ 0.213***	$-$ 6.012***
	(0.050)	(2.215)	(0.043)	(1.558)
Age squared	0.001***	0.053***	0.001***	0.035***
	(0.000)	(0.013)	(0.000)	(0.009)
Married	$-$ 0.151**	$-$ 6.600**	$-$ 0.002	$-$ 0.852
	(0.065)	(3.182)	(0.045)	(1.787)
Number of children	$-$ 0.037*	$-$ 1.574**	0.013	$-$ 0.093
	(0.019)	(0.775)	(0.013)	(0.435)
Household size	0.007	0.244	$-$ 0.005	0.891*
	(0.015)	(0.657)	(0.013)	(0.506)
Wave 3	$-$ 0.040	0.540	$-$ 0.099	$-$ 0.523
	(0.089)	(3.794)	(0.073)	(2.492)
Constant	0.139*	4.526	0.139**	2.905
	(0.075)	(3.235)	(0.065)	(2.184)
Observations	3218	3183	4391	4339
N (persons)	2243	2221	3078	3049

***$p<0.01$, **$p<0.05$, *$p<0.10$. Robust standard errors clustered at the individual level in parentheses. The FD estimates use the “reg, cluster(ID)” command using the model in first differences. Columns 1–2 of Panel A present estimates for the effect of daily caregiving on employment and work hours for males

$^{\dagger }$We use $y_{i(t-2)}$ and $y_{i(t-3)}$ as instruments for the lagged dependent variable ($\Delta y_{i(t-1)}$)

In Panel B of Table 6, we report (second-stage) Arellano Bond estimates for the effect of daily caregiving on employment and work hours by gender (again using the second and third lags of the dependent variable—$y_{i(t-s)}$ with $s=2,3$—as instruments for $\Delta y_{i(t-1)}$). In line with the results from static panel data models, daily caregiving does not significantly affect employment or work hours for males, but it is significant for females. For females, caregiving implies a 30.6% decrease of the employment probability and a 31.8% decrease of work hours (Panel B).^40^ As before, these effects are stronger than the corresponding effects obtained for the full sample (see Sects. 5.1–5.2). In line with the results reported in Sects. 5.1–5.2, the effect of daily caregiving on employment and work hours is stronger in the dynamic models than for the static models.

**Table 7:** The effects of caregiving on paid work by geographical region—FD estimates

	(1)	(2)	(3)	(4)
	Dependent variables			
	Employment	Employment	Work hours	Work hours
Informal caregiving	$-$ 0.003	–	$-$ 0.175	–
	(0.016)		(0.696)	
Informal caregiving * South Europe	$-$ 0.022	–	$-$ 0.944	–
	(0.022)		(0.987)	
Informal caregiving * Eastern Europe	$-$ 0.0001	–	0.386	–
	(0.023)		(1.005)	
Informal caregiving * Western Europe	0.001	–	$-$ 0.121	–
	(0.018)		(0.771)	
Daily caregiving	–	$-$ 0.070	–	$-$ 2.991
		(0.057)		(1.836)
Daily caregiving * South Europe	–	0.031	–	0.369
		(0.060)		(2.067)
Daily caregiving * Eastern Europe	–	0.032	–	1.466
		(0.061)		(2.072)
Daily caregiving * Western Europe	–	0.060	–	2.144
		(0.059)		(1.940)
Age	0.047***	0.047***	1.912***	1.928***
	(0.011)	(0.011)	(0.488)	(0.488)
Age squared	$-$ 0.0005***	$-$ 0.0005***	$-$ 0.018***	$-$ 0.018***
	(0.0001)	(0.0001)	(0.003)	(0.003)
Married (dummy)	$-$ 0.015	$-$ 0.015	$-$ 1.540**	$-$ 1.537**
	(0.014)	(0.014)	(0.632)	(0.631)
Number of children	$-$ 0.008*	$-$ 0.008*	$-$ 0.350*	$-$ 0.348*
	(0.005)	(0.005)	(0.181)	(0.181)
Household size	$-$ 0.005	$-$ 0.005	$-$ 0.057	$-$ 0.065
	(0.004)	(0.004)	(0.163)	(0.163)
Wave 3	$-$ 0.056***	$-$ 0.056***	$-$ 2.660***	$-$ 2.654***
	(0.014)	(0.014)	(0.589)	(0.589)
Wave 4	$-$ 0.057***	$-$ 0.057***	$-$ 2.543***	$-$ 2.534***
	(0.014)	(0.014)	(0.586)	(0.585)
Constant	$-$ 0.051***	$-$ 0.071***	$-$ 3.017***	$-$ 3.027***
	(0.015)	(0.018)	(0.768)	(0.768)
Observations	27,867	27,864	27,621	27,618
N (persons)	20,954	20,952	20,788	20,786
Adjusted $R^2$	0.010	0.010	0.010	0.011

***$p<0.01$, **$p<0.05$, *$p<0.10$. Robust standard errors clustered at the individual level in parentheses. The FD estimates use the “reg, cluster(ID)” command for the model in first differences. The baseline geographical region is Northern Europe (Sweden and Denmark)

### Heterogeneity Across Geographical Regions

Since European regions differ widely in terms of labor market attachment (see Table 2), formal care arrangements, and rates of informal (or daily) care provision (Table 3), it seems useful to investigate whether the effects of informal care on paid work differ across geographical regions. We generated dummies for Western Europe, Southern Europe and Eastern Europe; the baseline is Northern Europe.^41^ We interacted these variables with the aforementioned informal care dummies, and, using the static FD estimator, we regressed the paid work variables on informal caregiving (or daily caregiving) and interactions with geographical region dummies. The results of the (static) FD estimator are reported in Table 7. Even though there are large differences in terms of labor market attachment and rates of informal (or daily) care provision across geographical regions, the effects of informal caregiving and daily caregiving on paid work variables do not statistically significantly differ across geographical regions.

**Table 8:** The effects of caregiving on paid work by geographical region—AB estimates

	(1)	(2)	(3)	(4)
	Dependent variables			
	Employment	Employment	Work hours	Work hours
Employed$_{i,t-1}^{\dagger }$	0.509***	0.509***	–	–
	(0.026)	(0.026)		
Work hours$_{i,t-1}^{\dagger }$	–	–	0.482***	0.483***
			(0.028)	(0.028)
Informal caregiving	$-$ 0.037	–	$-$ 0.985	–
	(0.034)		(1.369)	
Informal caregiving * South Europe	0.007	–	$-$ 1.717	–
	(0.047)		(2.138)	
Informal caregiving * Eastern Europe	0.040	–	$-$ 1.261	–
	(0.097)		(4.736)	
Informal caregiving * Western Europe	0.033	–	0.055	–
	(0.039)		(1.584)	
Daily caregiving	–	$-$ 0.164	–	$-$ 5.102*
		(0.108)		(2.962)
Daily caregiving * South Europe	–	0.104	–	0.996
		(0.114)		(3.512)
Daily caregiving * Eastern Europe	–	$-\,0.035$	–	$-$ 1.368
		(0.164)		(6.649)
Daily caregiving * Western Europe	–	0.126	–	3.651
		(0.113)		(3.277)
Age	$-$ 0.236***	$-$ 0.234***	$-$ 7.419***	$-$ 7.373***
	(0.033)	(0.033)	(1.319)	(1.319)
Age squared	0.001***	0.001***	0.043***	0.043***
	(0.000)	(0.000)	(0.008)	(0.008)
Married	$-$ 0.052	$-$ 0.052	$-$ 2.674*	$-$ 2.638*
	(0.038)	(0.037)	(1.604)	(1.598)
Number of children	$-$ 0.008	$-$ 0.008	$-$ 0.696	$-$ 0.691
	(0.012)	(0.012)	(0.431)	(0.432)
Household size	0.001	0.001	0.585	0.575
	(0.010)	(0.010)	(0.411)	(0.412)
Wave 3	0.039	0.038	0.135	0.090
	(0.028)	(0.028)	(1.085)	(1.085)
Constant	0.183**	0.181**	4.008	3.863
	(0.076)	(0.076)	(2.920)	(2.920)
Observations	7609	7609	7522	7522
N (persons)	5321	5321	5270	5270

***$p<0.01$, **$p<0.05$, *$p<0.10$. Robust standard errors clustered at the individual level in parentheses. The baseline geographical region is Northern Europe (Sweden and Denmark)

$^{\dagger }$We use $y_{i(t-2)}$ and $y_{i(t-3)}$ as instruments for the lagged dependent variable ($\Delta y_{i(t-1)}$)

We also replicated the previous analysis using the Arellano Bond estimator, see Table 8.^42^ The interaction terms described above were statistically insignificant, implying that the effects of informal caregiving and daily caregiving on employment and work hours are not statistically different across European regions.

## Conclusion 

We have analyzed the causal effects of informal caregiving and daily caregiving to elderly parents (biological parents, parents-in-law, stepparents) and grandparents on employment and work hours among the 50+ population in 15 European countries, using longitudinal data that cover the time period 2004–2013. We have focused on static and dynamic panel data models that allow for potential endogeneity of the main explanatory variable of interest, exploiting heteroscedasticity-based instruments and instrumental variables that have been introduced in the existing literature. From a methodological point of view, a main finding is that the results are sensitive to the chosen specification. In particular, panel data models that allow for fixed effects lead to different results than models that do not account for correlation between individual effects and the informal care variable that is the main explanatory variable of interest. But even when fixed effects are incorporated, the size and significance level of the effects of (daily) informal caregiving depend on whether a static or dynamic model is used and on whether an immediate reverse causal effect of paid work on informal care is allowed for. This can explain a large part of the variation in existing findings in the extensive empirical literature on the topic. It emphasizes the importance of selecting models that are supported by the data.

Using models that pass tests for misspecification, we find that informal caregiving at low intensity does not significantly affect the probability of being employed or hours of paid work. On the other hand, we find negative effects of daily or almost daily caregiving on both the employment probability and weekly hours of paid work. Moreover, these negative effects of daily caregiving are much stronger for females than for males. Using our preferred models for employment and work hours, in which the caregiving variables appear to be exogenous, we find that daily caregiving decreases the probability of being employed by 6.5-percentage-points (22.0%) and reduces hours of paid work by almost 28%. These effects are substantial and have important implications, even though the share of individuals who provide daily caregiving is limited - around 2% of all observations on males and 4% of all observations on females. In spite of the many differences across European regions, we find no evidence of heterogeneity of the effects of informal or daily caregiving across the European regions that we consider (Western, Eastern, Southern or Northern Europe).

The policy implications of negative effects of caregiving have been extensively discussed in the existing literature. See, e.g., OECD (2013), Heitmueller (2007) and Colombo et al. (2011). Due to population ageing and the increasing costs of formal long-term care provision, many governments try to shift part of the responsibility for long-term care of the elderly to children or other family members, increasing the demand for informal care provision. Negative spill-over effects on (for example) labor supply of care providers should be taken into account when evaluating this type of policy. We find no evidence that low-intensity informal care would have such a negative effect. If, however, formal care possibilities would become so scarce or expensive that daily or almost daily informal care needs to be provided, then the large negative effects on employment and hours of paid work that we find would be a source of concern. This conclusion was also drawn by Heitmueller (2007) for his analysis of UK data. We find essentially the same result for a large set of European countries, with very different institutional arrangements for formal care of the elderly and with very different labor market institutions. Moreover, we find that daily caregiving in particular has negative effects for women, hampering policies that are aimed at increasing the labor force participation of women in the age group 50 and older.

Based on these results, specific policies that reduce the need for daily caregiving to elderly parents deserve serious consideration. One possibility is to create opportunities for substitution, e.g. vouchers or cash benefits that can be used to outsource part of the caregiving duties to specialized personnel. Alternatively, counseling or training sessions for intensive caregivers might be useful. Whether the latter policies are really effective is an open research question. Moreover, we have only considered the potentially negative effects on labor supply and other (negative or positive) side-effects should be considered also when evaluating this type of policies. The existing literature shows that other negative effects may well exist, see for example the evidence of negative effects on (mental) health in Coe and van Houtven (2009) for the US. Whether other effects of (daily) caregiving play a similar role in Europe remains to be investigated.

### Electronic supplementary material

Below is the link to the electronic supplementary material.

Supplementary material 1 (pdf 270 KB)

## Appendix

**Table 9:** Countries included in the longitudinal dataset

	Geographical region	Wave 1	Wave 2	Wave 4	Wave 5
*Countries observed in all waves (waves 1,2,4,5)*					
Denmark	Northern Europe	X	X	X	X
Sweden	Northern Europe	X	X	X	X
Austria	Western Europe	X	X	X	X
Belgium	Western Europe	X	X	X	X
France	Western Europe	X	X	X	X
Germany	Western Europe	X	X	X	X
Netherlands	Western Europe	X	X	X	X
Switzerland	Western Europe	X	X	X	X
Italy	Southern Europe	X	X	X	X
Spain	Southern Europe	X	X	X	X
*Countries observed in few waves*					
Greece	Southern Europe	X	X		
Czech Republic	Eastern Europe		X	X	X
Estonia	Eastern Europe			X	X
Poland	Eastern Europe		X	X	
Slovenia	Eastern Europe			X	X

Data for 10 nations is available for all waves used in analysis (i.e, waves 1–2 and 4–5). Data for Greece, Czech Republic, Estonia, Poland and Slovenia is available only for some waves

**Table 10:** Descriptive statistics for control variables

	Description	Obs.	Mean	SD	Min	Max
*Panel A: Males and females*						
Age	Age in years	27,867	61.46	4.90	52	70
Age squared	Age squared	27,867	3801.14	601.14	2704	4900
Married	1 if married or partnered	27,867	0.64	0.48	0	1
Number of children	Total number of living children	27,867	2.06	1.29	0	16
Household size	Number of persons living in the household	27,867	2.12	1.01	1	10
*Panel B: Males*						
Age	Age in years	11,791	61.54	4.92	52	70
Age squared	Age squared	11,791	3810.93	604.20	2704	4900
Married	1 if married or partnered	11,791	0.71	0.45	0	1
Number of children	Total number of living children	11,791	2.02	1.33	0	16
Household size	Number of persons living in the household	11,791	2.22	1.03	1	10
*Panel C: Females*						
Age	Age in years	16,076	61.40	4.88	52	70
Age squared	Age squared	16,076	3793.97	598.81	2704	4900
Married	1 if married or partnered	16,076	0.59	0.49	0	1
Number of children	Total number of living children	16,076	2.08	1.26	0	13
Household size	Number of persons living in the household	16,076	2.04	0.98	1	9

SHARE, 2004–2013, ages 50–70. Estimation sample for static model in first differences (Table 5)

**Table 11:** Regressions of employment status on both non-daily and daily caregiving

	(1)	(2)	(3)
	OLS	FD	AB
	Dependent variable		
	Employment	Employment	Employment
Non-daily caregiving	0.106***	0.001	$-$ 0.001
	(0.010)	(0.007)	(0.018)
Daily caregiving	$-$ 0.094***	$-$ 0.027**	$-$ 0.065**
	(0.015)	(0.011)	(0.025)
Employment$$_{i,t-1} $$	–	–	0.509***
			(0.026)
Individual FE?	No	Yes	Yes
Wave FE?	Yes	Yes	Yes
Controls?$^{\dagger }$	All	All	All
Observations	27,864	27,864	7609
N (persons)	20,952	20,952	5321
Adjusted $R^2$	0.287	0.010	–
*Test results*			
F-statistic (excluded IVs)	–	–	1037.715
Hansen J test (p-value)	–	–	0.373
Hausman test (p-value)	–	–	0.000
*Test of serial correlation of the error term:* $\Delta \epsilon _{it} = \rho \Delta \epsilon _{i,t-1} + error_{it}$			
p-value (test: $\widehat{\rho } =-0.5$)	–	–	0.518

***$p<0.01$, **$p<0.05$, *$p<0.10$. Robust standard errors clustered at the individual level in parentheses. “OLS”, “FD”, “AB” refer to (pooled) ordinary least squares, first difference, and Arellano Bond estimators, respectively

$^{\dagger }$Controls include age, age squared, marital status (a dummy variable equal to 1 for married persons, 0 otherwise), number of children, and household size

**Table 12:** Regressions of work hours on both non-daily and daily caregiving

	(1)	(2)	(3)
	OLS	FD	AB
	Dependent variable		
	Work hours	Work hours	Work hours
Non-daily caregiving	3.792***	0.060	$-$ 0.669
	(0.405)	(0.291)	(0.706)
Daily caregiving	$-$ 4.305***	$-$ 1.540***	$-$ 3.045***
	(0.575)	(0.454)	(1.073)
Work hours$$_{i,t-1} $$	–	–	0.482***
			(0.028)
Individual FE?	No	Yes	Yes
Wave FE?	Yes	Yes	Yes
Controls?$^{\dagger }$	All	All	All
Observations	27,618	27,618	7522
N (persons)	20,786	20,786	5270
Adjusted $R^2$	0.254	0.011	–
Test results			
F-statistic (excluded IVs)	–	–	839.400
Hansen J test (p-value)	–	–	0.897
Hausman test (p-value)	–	–	0.000
Test of serial correlation of the error term: $\Delta \epsilon _{it} = \rho \Delta \epsilon _{i,t-1} + error_{it}$			
p-value (test: $\widehat{\rho } =-0.5$)	–	–	0.027

***$p<0.01$, **$p<0.05$, *$p<0.10$. Robust standard errors clustered at the individual level in parentheses. “OLS”, “FD”, “AB” refer to (pooled) ordinary least squares, first difference, and Arellano Bond estimators, respectively

$^{\dagger }$Controls include age, age squared, marital status (a dummy variable equal to 1 for married persons, 0 otherwise), number of children, and household size

## Footnotes

1 Moreover, most elderly care-receivers receive help from their adult children (Coe and van Houtven 2009).

2 It can be seen as a limitation that SHARE is restricted to 50+ individuals, but the age group 50–70 is the group for which providing informal care to elderly parents is most prevalent.

3 This is not the complete list of instrumental variables that were used in previous studies; see Sect. 2.

4 We distinguish Northern Europe, Western Europe, Eastern Europe, and Southern Europe.

5 Data for wave 6 of SHARE are not used in the analysis, since they were not available at the start of the project. For more information on the data collection, see http://www.share-project.org/data-documentation/waves-overview.html.

6 This excludes Ireland, Luxembourg, Hungary and Portugal.

7 We do not consider wage rates since they can only be computed for the first two waves of SHARE; see Flores and Kalwij (2013) (page 7).

8 For simplicity and ease of comprehension, Table 2 reports summary statistics on the number of work hours conditional on being employed in the current wave, rather than summary statistics for the actual variable for work hours that includes zeros.

9 Informal care given to other sick or disabled individuals is not considered in this study, since data on this are available only for waves 1 and 2. Data on co-residential care to “parents” is not considered, since they do not contain the same information on frequency and type of care provided.

10 In waves 1–2, respondents are asked the following question: “In the last twelve months, have you personally given any kind of help listed on this card to a family member from outside the household, a friend or neighbour?” If the answer is positive, then respondents are asked: “Which types of help have you given to this person in the last twelve months? (a) Personal care, e.g. dressing, (b) practical household help, e.g. shopping, (c) help with paperwork, e.g. filling out forms”.

11 In waves 4 and 5, respondents are asked the following question: “In the last twelve months, have you personally given personal care or practical household help to a family member living outside your household, a friend or neighbour?”

12 We also experimented with different measures of intensive caregiving, but found that the only distinction that mattered was (almost) daily frequency or less often than daily frequency.

13 We do not control for household income since this is endogenous to employment status. Differently from Ciani (2012) and Heitmueller (2007) but similarly to Bolin et al. (2008), we do not control for work disability, since in SHARE, work disability questions are only asked to non-workers.

14 For static panel data models, we also report results from pooled OLS estimations (with wave fixed effects) in order to provide correlational evidence.

15 Since the dynamic panel data models are estimated in first differences (see below), for ease of comparability we also estimate the static panel data model (1) in first differences rather than using the within group transformation.

16 Following Arellano and Bond (1991), missing values for instruments $y_{is}$ are replaced by zeros.

17 We considered adding moments in levels (“System GMM”; see Blundell and Bond 1998) but this either gave virtually identical results or model specifications that were rejected by standard specification tests. Moreover, the fact that the estimated coefficient on the state dependence variable is always much lower than 1, suggests that moments in levels are not necessary. System GMM estimates are available from the authors.

18 This requires the following steps: (1) Estimate the dynamic panel data model in first differences (see eq. (3)), using $y_{i(t-2)}$ and $y_{i(t-3)}$ as instruments for $\Delta y_{i(t-1)}$. (2) Generate the residuals ($\widehat{\Delta \epsilon _{it}}$) from step 1, and compute the lagged residuals ($\widehat{\Delta \epsilon _{i(t-1)}}$). (3) Perform OLS of $\widehat{\Delta \epsilon _{it}}$ on $\widehat{\Delta \epsilon _{i(t-1)}}$. If the error term in levels is not serially correlated, then the coefficient should be equal to $-0.5$. Values larger than $-0.5$ indicate that the error term is *positively serially correlated*. Values of $\widehat{\rho }$ smaller than $-0.5$ indicates that the error term is *negatively serially correlated*. (4) Test whether $\widehat{\rho }$ is equal to $-0.5$ using a Wald test.

19 Column 3 of Table 4 shows that the respondent’s father is dead for 89.7% of observations (24,988 out of 27,867 observations), and we find that the respondent’s mother is dead for 71.2% of observations (19,847 out of 27,867 observations).

20 The F-statistic for “distance from the father’s residence” is 3.87 when the instrumented variable is “informal caregiving”, and 0.00 when the instrumented variable is “daily caregiving”.

21 Complete regression results for OLS and FD estimations are reported in Table 13 in the electronic supplementary materials.

22 Furthermore, the first-stage coefficients on the instrumental variables “mother is dead”, “father is dead”, “mother’s bad health”, “father’s bad health” and “distance from mother’s residence” are as expected (see Column 1 of Table  14 (electronic supplementary materials) for the first-stage estimates).

23 See Column 3 of Table 14 (electronic supplementary materials for first-stage estimates.

24 The second-stage coefficient on daily caregiving from the FDIV estimation is statistically insignificant, but it must be stressed that the standard errors from FDIV estimations are at least 4 times larger than the standard errors from FD estimations; see Columns 3–6 of Table 4.

25 First-stage estimates for dynamic models are reported in Column 1 and Column 3 of Table 15 (electronic supplementary materials). In Column 1 of Table 15 (electronic supplementary materials), informal caregiving is included in the set of independent variables. In Column 3 of Table 15 (electronic supplementary materials), daily caregiving is included in the set of independent variables.

26 In the case of dynamic panel data models, we tested the exogeneity of the caregiving variables as follows. We used the eight instrumental variables specified in Sect. 4.1 for the caregiving variables, and $y_{i(t-2)}$ and $y_{i(t-3)}$ as the instruments for the state dependence variable in differences ($\Delta y_{i(t-1)}$). We then tested the exogeneity of each caregiving variable using the Hausman test exclusively for the caregiving variable. The null hypothesis of exogeneity of the caregiving variable is not rejected at the 5% level, both for informal caregiving and for daily caregiving. These estimates are not reported and are available from the authors.

27 I.e., $$-\,0.065/0.295= -\,0.220$$.

28 See Sect. 3 for the definition of the dummy variables for informal care provision and daily care provision.

29 These estimates are not reported and are available from the authors.

30 In the case of the AB estimator, we use the second and third lag of employment status as the instrumental variables for lagged employment status.

31 Complete regression results for OLS and FD estimations are reported in Table 16 (electronic supplementary materials).

32 I.e., $$-1.55/13.19=-\,0.118$$.

33 The average number of paid work hours is 13.19. Thus, the estimated (second-stage) coefficient on informal caregiving (0.205) implies that informal caregiving has a very small (and insignificant) effect on the number of paid work hours.

34 See Column 3 of Table 17 (electronic supplementary materials) for the first-stage estimates.

35 Corresponding first-stage estimates are reported in Columns 1 (using any caregiving) and 3 (using daily caregiving) of Table 18 (electronic supplementary materials).

36 See Footnote 26.

37 The average of work hours for the dynamic model is 10.452. Thus, daily caregiving implies a 27.7% decrease of work hours (i.e., -2.902/10.452).

38 I.e., $$-\,2.400/10.452 = -\,0.2296$$.

39 I.e., $$-\,0.034/0.324=-\,0.105$$; $$-\,1.410/10.804= -\,0.131$$.

40 I.e., $$-\,0.081/0.265=-\,0.306$$; $$-\,2.640/8.296= -\,0.318$$.

41 Western Europe: Austria, Belgium, France, Germany, Netherlands, Switzerland. Southern Europe: Greece, Italy, Spain. Eastern Europe: Czech Republic, Estonia, Poland, Slovenia. Northern Europe: Denmark, Sweden.

42 First-stage estimates are not reported and are available from the authors.
