# Supplementary material for: Informal Caregiving, Employment Status and Work Hours of the 50+ Population in Europe
Source: Economist (Leiden). 2018 May 31;166(3):363–96. doi: 10.1007/s10645-018-9323-1 (PMC6434966; doi:10.1007/s10645-018-9323-1)
Supplement: Supplementary file 1 — Supplementary material 1 (pdf 270 KB) [file 10645_2018_9323_MOESM1_ESM.pdf]

Online Supplementary Material for Informal Caregiving, Employment Status and Work Hours of the 50+ Population in Europe

Nicola Ciccarelli*, Arthur Van Soest†

*CentER and Department of Econometrics and Operations Research, Tilburg University, P.O. Box 90153, 5000 LE Tilburg, The Netherlands. Phone: +31 13 466 3254, E-mail: ciccarelli.nicola@gmail.com.

†CentER and Department of Econometrics and Operations Research, Tilburg University, P.O. Box 90153, 5000 LE Tilburg, The Netherlands.

Table 13: The Effects of Caregiving on Employment

	(1) OLS	(2) OLS	(3) FD	(4) FD
	Dependent variable: employment			
Informal caregiving	0.057***	-	-0.005	-
	(0.008)		(0.006)	
Daily caregiving	-	-0.107***	-	-0.027**
		(0.015)		(0.011)
Age	-0.133***	-0.131***	0.047***	0.047***
	(0.013)	(0.013)	(0.011)	(0.011)
Age squared	0.001***	0.001***	-0.0005***	-0.0005***
	(0.0001)	(0.0001)	(0.0001)	(0.0001)
Married (dummy)	0.007	0.010	-0.015	-0.015
	(0.007)	(0.007)	(0.014)	(0.014)
Number of children	0.0004	5.99e-06	-0.008*	-0.008*
	(0.0022)	(0.002)	(0.005)	(0.005)
Household size	-0.010***	-0.010***	-0.005	-0.005
	(0.003)	(0.003)	(0.004)	(0.004)
Wave 3	0.035***	0.039***	-0.057***	-0.057***
	(0.006)	(0.006)	(0.014)	(0.014)
Wave 4	0.027***	0.032***	-0.057***	-0.057***
	(0.006)	(0.006)	(0.014)	(0.014)
Constant	6.005***	5.978***	-0.052***	-0.052***
	(0.410)	(0.411)	(0.015)	(0.015)
**Individual FE?**	**NO**	**NO**	**YES**	**YES**
Observations	27,867	27,864	27,867	27,864
N (persons)	20,954	20,952	20,954	20,952
Adjusted R^2	0.283	0.283	0.010	0.010

Notes: *** $p < 0.01$, ** $p < 0.05$, * $p < 0.10$. Robust standard errors clustered at the individual level in parentheses. ‘ “OLS” refers to the (pooled) ordinary least squares estimator; “FD” refers to the first difference estimator. The FD estimates use the “reg, cluster(ID)” command for the model in first differences.

Table 14: The Effects of Caregiving on Employment – First-stage and Second-stage FDIV Estimates

	(1) FDIV First stage	(2) FDIV Second stage	(3) FDIV First stage	(4) FDIV Second stage
	Dependent variables			
	Informal caregiving	Employment	Daily caregiving	Employment
Heteroscedasticity IV (age)	-0.058***	-	-0.223***	-
	(0.020)		(0.037)	
Heteroscedasticity IV (h. size)	-0.094***	-	-0.020	-
	(0.023)		(0.040)	
Heteroscedasticity IV (wave 2)	-0.188***	-	-0.477***	-
	(0.031)		(0.067)	
Father is dead	-0.038*	-	0.005	-
	(0.020)		(0.011)	
Mother is dead	-0.080***	-	-0.0004	-
	(0.015)		(0.008)	
Father's bad health	0.042***	-	0.016***	-
	(0.010)		(0.005)	
Mother's bad health	0.039***	-	0.010	-
	(0.015)		(0.008)	
Distance from mother (dummy)	0.057***	-	0.036***	-
	(0.014)		(0.010)	
Informal caregiving	-	0.028	-	-
		(0.038)		
Daily caregiving	-	-	-	-0.024
				(0.045)
Age	0.033***	0.046***	0.007	0.047***
	(0.012)	(0.011)	(0.007)	(0.011)
Age squared	-0.000***	-0.000***	-0.000**	-0.000***
	(0.000)	(0.000)	(0.000)	(0.000)
Married (dummy)	0.006	-0.016	-0.001	-0.015
	(0.014)	(0.014)	(0.007)	(0.014)
Number of children	0.001	-0.009*	0.001	-0.008*
	(0.004)	(0.005)	(0.002)	(0.005)
Household size	0.002	-0.005	-0.002	-0.005
	(0.004)	(0.004)	(0.002)	(0.004)
Wave 3	0.040***	-0.058***	0.023***	-0.057***
	(0.015)	(0.014)	(0.008)	(0.014)
Wave 4	0.058***	-0.059***	0.029***	-0.057***
	(0.015)	(0.014)	(0.008)	(0.014)
Constant	0.006	-0.052***	0.004	-0.052***
	(0.017)	(0.015)	(0.009)	(0.015)
**Individual FE?**	**Yes**	**Yes**	**Yes**	**Yes**
Observations	27,867	27,867	27,864	27,864
N (persons)	20,954	20,954	20,952	20,952
F-statistic (excluded instruments)†	-	28.676	-	11.050
p-value	-	0.0000	-	0.0000
Hansen J statistic	-	9.494	-	11.625
p-value	-	0.219	-	0.114
Hausman test statistic	-	0.922	-	0.127
p-value	-	0.3370	-	0.721

Notes: *** $p < 0.01$, ** $p < 0.05$, * $p < 0.10$. Robust standard errors clustered at the individual level in parentheses. The FDIV estimates use the "ivreg2, cluster(ID)" command for the model in first differences. The F-statistic is a test of joint significance of the excluded instrumental variables. Under the null hypothesis of the Hansen test, the over-identifying restrictions are valid. Under the null hypothesis of the Hausman test, the instrumented variable is exogenous.
†The F-statistic reported in this table is the Kleibergen-Paap rank Wald F statistic, which is valid under heteroscedasticity and arbitrary correlation of the error term.

Table 15: The Effects of Caregiving on Employment – First and Second Stage AB (Arellano Bond) Estimates

	(1) AB First stage	(2) AB Second stage	(3) AB First stage	(4) AB Second stage
	Dependent variables			
	ΔEmployment$_{i,t-1}$	ΔEmployment$_{i,t}$	ΔEmployment$_{i,t-1}$	ΔEmployment$_{i,t}$
IV #1 (Employment$_{i,t-2}$)	-0.423***	-	-0.423***	-
	(0.010)		(0.010)	
IV #2 (Employment$_{i,t-3}$)	-0.018	-	-0.018	-
	(0.015)		(0.015)	
ΔEmployment$_{i(t-1)}$	-	0.509***	-	0.509***
		(0.026)		(0.026)
ΔInformal caregiving$_{it}$	0.017	-0.016	-	-
	(0.011)	(0.016)		
ΔDaily caregiving$_{it}$	-	-	0.015	-0.065**
			(0.019)	(0.025)
ΔAge$_{it}$	0.612***	-0.235***	0.611***	-0.234***
	(0.027)	(0.033)	(0.027)	(0.033)
ΔAge squared$_{it}$	-0.005***	0.001***	-0.005***	0.001***
	(0.000)	(0.000)	(0.000)	(0.000)
ΔMarried$_{it}$	0.030	-0.053	0.030	-0.052
	(0.026)	(0.038)	(0.026)	(0.037)
ΔNumber of children$_{it}$	-0.002	-0.008	-0.002	-0.007
	(0.008)	(0.012)	(0.008)	(0.012)
ΔHousehold size$_{it}$	0.000	0.001	0.001	0.001
	(0.007)	(0.010)	(0.007)	(0.010)
ΔWave 4$_{it}$	0.040	-0.077	0.039	-0.076
	(0.039)	(0.057)	(0.039)	(0.057)
Constant	-0.003	0.220**	-0.001	0.218**
	(0.072)	(0.104)	(0.072)	(0.104)
**Individual FE?**	**Yes**	**Yes**	**Yes**	**Yes**
Observations	7,609	7,609	7,609	7,609
N (persons)	5,321	5,321	5,321	5,321
F-statistic (excluded instruments)†	-	1037.908	-	1037.175
p-value	-	0.0000	-	0.0000
Hansen J statistic	-	0.776	-	0.797
p-value	-	0.378	-	0.372
Hausman test statistic	-	1216.053	-	1217.407
p-value	-	0.000	-	0.000
Test of serial correlation of the error term in differences: $\Delta\epsilon_{it} = \rho\Delta\epsilon_{i,t-1} + error_{it}$††				
$\widehat{\rho}$	-	-0.509***	-	-0.509***
	-	(0.014)	-	(0.014)
p-value ($\widehat{\rho} = -0.5$)††	-	0.502	-	0.519

Notes: *** $p < 0.01$, ** $p < 0.05$, * $p < 0.10$. Robust standard errors clustered at the individual level in parentheses. The FDIV estimates use the "ivreg2, cluster(ID)" command for the model in first differences. The F-statistic is a test of joint significance of the excluded instrumental variables. Under the null hypothesis of the Hansen test, the over-identifying restrictions are valid. Under the null hypothesis of the Hausman test, the instrumented variable is exogenous.
†The F-statistic reported in this table is the Kleibergen-Paap rank Wald F statistic, which is valid under heteroscedasticity and arbitrary correlation of the error term.
††Under the null that the error terms in levels (ϵ_{it}) are not serially correlated, $\widehat{\rho}$ from the regression $\Delta\epsilon_{it} = \rho\Delta\epsilon_{i,t-1} + error_{it}$ should not differ significantly from -0.5 (see Wooldridge (2002), Section 10.6.3).

Table 16: The Effects of Caregiving on Paid Work Hours

	(1)	(2)	(3)	(4)
	Dependent variable: work hours			
Informal caregiving	1.829***	-	-0.292	-
	(0.353)		(0.262)	
Daily caregiving	-	-4.757***	-	-1.553***
		(0.574)		(0.448)
Age	-5.728***	-5.636***	1.918***	1.931***
	(0.564)	(0.565)	(0.489)	(0.488)
Age squared	0.031***	0.030***	-0.018***	-0.018***
	(0.005)	(0.005)	(0.003)	(0.003)
Married (dummy)	-0.146	-0.056	-1.543**	-1.545**
	(0.284)	(0.285)	(0.632)	(0.631)
Number of children	-0.072	-0.089	-0.351*	-0.348*
	(0.094)	(0.095)	(0.181)	(0.181)
Household size	-0.024	-0.020	-0.059	-0.064
	(0.151)	(0.151)	(0.163)	(0.163)
Wave 3	0.967***	1.116***	-2.675***	-2.664***
	(0.247)	(0.246)	(0.589)	(0.589)
Wave 4	0.868***	1.090***	-2.558***	-2.543***
	(0.239)	(0.238)	(0.586)	(0.585)
Constant	247.820***	246.150***	-2.177***	-2.186***
	(17.437)	(17.460)	(0.649)	(0.648)
**Individual FE?**	**NO**	**NO**	**YES**	**YES**
Observations	27,621	27,618	27,621	27,618
N (persons)	20,788	20,786	20,788	20,786
Adjusted R^2	0.250	0.251	0.010	0.011

Notes: *** $p < 0.01$, ** $p < 0.05$, * $p < 0.10$. Robust standard errors clustered at the individual level in parentheses. "OLS" refers to the (pooled) ordinary least squares estimator; "FD" refers to the first difference estimator. The FD estimates use the "reg, cluster(ID)" command for the model in first differences.

Table 17: The Effects of Caregiving on Work Hours – First-stage and Second-stage FDIV Estimates

	(1) FDIV First stage	(2) FDIV Second stage	(3) FDIV First stage	(4) FDIV Second stage
	Dependent variables			
	Informal caregiving	Work hours	Daily caregiving	Work hours
Heteroscedasticity IV (age)	-0.061***	-	-0.226***	-
	(0.020)		(0.037)	
Heteroscedasticity IV (h. size)	-0.097***	-	-0.019	-
	(0.023)		(0.040)	
Heteroscedasticity IV (wave 2)	-0.195***	-	-0.494***	-
	(0.031)		(0.068)	
Father is dead	-0.041**	-	0.004	-
	(0.020)		(0.011)	
Mother is dead	-0.078***	-	0.002	-
	(0.015)		(0.008)	
Mother's bad health	0.042***	-	0.015***	-
	(0.010)		(0.005)	
Father's bad health	0.038**	-	0.009	-
	(0.015)		(0.008)	
Distance from mother (dummy)	0.057***	-	0.036***	-
	(0.014)		(0.010)	
Informal caregiving	-	0.205	-	-
		(1.574)		
Daily caregiving	-	-	-	-0.758
				(1.886)
Age	0.035***	1.897***	0.006	1.920***
	(0.012)	(0.494)	(0.007)	(0.489)
Age squared	-0.000***	-0.018***	-0.000**	-0.018***
	(0.000)	(0.003)	(0.000)	(0.003)
Married	0.009	-1.550**	-0.001	-1.546**
	(0.014)	(0.632)	(0.007)	(0.631)
Number of children	0.001	-0.352*	0.001	-0.350*
	(0.004)	(0.181)	(0.002)	(0.181)
Household size	0.002	-0.060	-0.002	-0.062
	(0.004)	(0.163)	(0.002)	(0.163)
Wave 3	0.040***	-2.690***	0.024***	-2.679***
	(0.015)	(0.590)	(0.008)	(0.590)
Wave 4	0.059***	-2.587***	0.030***	-2.566***
	(0.015)	(0.590)	(0.008)	(0.587)
Constant	0.005	-2.176***	0.004	-2.188***
	(0.017)	(0.649)	(0.009)	(0.648)
**Individual FE?**	**Yes**	**Yes**	**Yes**	**Yes**
Observations	27,621	27,621	27,618	27,618
N (persons)	20,788	20,788	20,786	20,786
F-statistic (excluded instruments)†	-	28.908	-	10.979
p-value	-	0.0000	-	0.0000
Hansen J statistic	-	7.020	-	7.870
p-value	-	0.426	-	0.344
Hausman test statistic	-	0.149	-	0.364
p-value	-	0.699	-	0.546

Notes: *** $p < 0.01$, ** $p < 0.05$, * $p < 0.10$. Robust standard errors clustered at the individual level in parentheses. The FDIV estimates use the "ivreg2, cluster(ID)" command for the model in first differences. The F-statistic is a test of joint significance of the excluded instrumental variables. Under the null hypothesis of the Hansen test, the over-identifying restrictions are valid. Under the null hypothesis of the Hausman test, the instrumented variable is exogenous.
†The F-statistic reported in this table is the Kleibergen-Paap rank Wald F statistic, which is valid under heteroscedasticity and arbitrary correlation of the error term.

Table 18: The Effects of Caregiving on Work Hours – First and Second Stage AB (Arellano Bond) Estimates

	(1) AB First stage	(2) AB Second stage	(3) AB First stage	(4) AB Second stage
	Dependent variables			
	ΔWork hours$_{i,t-1}$	ΔWork hours$_{it}$	ΔWork hours$_{i,t-1}$	ΔWork hours$_{it}$
IV # 1 (Work hours$_{i,t-2}$)	-0.431***	-	-0.431***	-
	(0.011)		(0.011)	
IV # 1 (Work hours$_{i,t-3}$)	-0.001	-	-0.001	-
	(0.017)		(0.017)	
ΔWork hours$_{i(t-1)}$	-	0.482***	-	0.483***
		(0.028)		(0.028)
ΔInformal caregiving$_{it}$	1.039**	-1.218*	-	-
	(0.446)	(0.636)		
ΔDaily caregiving$_{it}$	-	-	0.877	-2.902***
			(0.764)	(1.057)
ΔAge$_{it}$	22.349***	-7.433***	22.331***	-7.382***
	(1.090)	(1.320)	(1.090)	(1.320)
Δ Age squared$_{it}$	-0.174***	0.043***	-0.174***	0.043***
	(0.007)	(0.008)	(0.007)	(0.008)
Δ Married$_{it}$	1.067	-2.689*	1.055	-2.650*
	(1.056)	(1.606)	(1.056)	(1.604)
Δ Number of children$_{it}$	0.117	-0.697	0.118	-0.689
	(0.296)	(0.431)	(0.297)	(0.432)
Δ Household size$_{it}$	-0.443	0.590	-0.441	0.583
	(0.295)	(0.411)	(0.295)	(0.411)
Δ Wave 4$_{it}$	0.423	-0.291	0.350	-0.223
	(1.583)	(2.172)	(1.583)	(2.172)
Constant	0.794	4.189	0.953	4.029
	(2.901)	(3.986)	(2.903)	(3.986)
**Individual FE?**	**Yes**	**Yes**	**Yes**	**Yes**
Observations	7,522	7,522	7,522	7,522
N (persons)	5,270	5,270	5,270	5,270
F-statistic (excluded instruments)[†]	-	840.009	-	839.039
p-value	-	0.0000	-	0.0000
Hansen J statistic		0.015		0.014
p-value		0.904		0.907
Hausman test statistic	-	846.445	-	847.650
p-value	-	0.000	-	0.000
Test of serial correlation of the error term in differences: $\Delta\epsilon_{it} = \rho\Delta\epsilon_{i,t-1} + error_{it}$[††]				
$\widehat{\rho}$	-	-0.467***	-	-0.467***
		(0.015)		(0.015)
p-value ($\widehat{\rho} = -0.5$)[††]	-	0.029	-	0.027

Notes: *** $p < 0.01$, ** $p < 0.05$, * $p < 0.10$. Robust standard errors clustered at the individual level in parentheses. The FDIV estimates use the "ivreg2, cluster(ID)" command for the model in first differences. The F-statistic is a test of joint significance of the excluded instrumental variables. Under the null hypothesis of the Hansen test, the over-identifying restrictions are valid. Under the null hypothesis of the Hausman test, the instrumented variable is exogenous.

[†]The F-statistic reported in this table is the Kleibergen-Paap rank Wald F statistic, which is valid under heteroscedasticity and arbitrary correlation of the error term.

[††]Under the null that the error terms in levels (ϵ_{it}) are not serially correlated, $\widehat{\rho}$ from the regression $\Delta\epsilon_{it} = \rho\Delta\epsilon_{i,t-1} + error_{it}$ should not differ significantly from -0.5 (see Wooldridge (2002), Section 10.6.3).
